# A Pilot Study Comparing the Efficacy, Fidelity, Acceptability, and Feasibility of Telehealth and Face-to-Face Creative Movement Interventions in Children with Autism Spectrum Disorder

**DOI:** 10.1089/tmr.2023.0061

**Published:** 2024-03-21

**Authors:** Wan-Chun Su, Corina Cleffi, Sudha Srinivasan, Anjana Bhat

**Affiliations:** ^1^ Department of Physical Therapy, University of Delaware, Newark, Delaware, USA.; ^2^ Biomechanics & Movement Science Program, University of Delaware, Newark, Delaware, USA.; ^3^ Physical Therapy Program, Department of Kinesiology, University of Connecticut, Storrs, Connecticut, USA.; ^4^ Institute for Health, Intervention, and Policy, University of Connecticut, Storrs, Connecticut, USA.; ^5^ The Connecticut Institute for the Brain and Cognitive Sciences, University of Connecticut, Storrs, Connecticut, USA.; ^6^ Department of Psychological and Brain Sciences, University of Delaware, Newark, Delaware, USA.

**Keywords:** autism spectrum disorder, telehealth, creative movement, stakeholders, motor performance

## Abstract

**Aims:**

We compared the efficacy, fidelity, acceptability, and feasibility of a creative movement (CM) intervention for children with autism spectrum disorder (ASD), delivered face-to-face (F2F) or through telehealth (TH).

**Methods:**

Fifteen children with ASD received the CM intervention F2F or through TH. Motor assessments were used to evaluate effects of F2F and TH interventions on children's motor skills, while video coding was used to assess affect, socially directed verbalization, interpersonal synchrony, and motor coordination during training. Stakeholder feedback and training fidelity data on the intervention were also collected.

**Results:**

Children in both subgroups showed similar baseline performance and training-related improvements in motor skills, positive/interested affect, socially directed verbalization, interpersonal synchrony, and dual/multilimb coordination. Parents in the TH subgroup considered the intervention feasible and acceptable; however, they reported greater effort to supervise and redirect their child's attention compared to the F2F subgroup. Trainers for the TH subgroup reported more communication difficulties, technological issues, and longer session lengths, but found greater parental involvement compared to the F2F subgroup.

**Conclusions:**

CM interventions are consistent, acceptable, feasible, and effective in improving social, behavioral-affective, and motor skills of children with ASD, regardless of the method of delivery. Clinicians should make efforts to reduce communication/technological issues and parental burden when delivering CM interventions through TH. ClinicalTrials.Gov Study ID-NCT04258254.

## Introduction

Autism spectrum disorder (ASD) is a multisystem disorder affecting children's social communication, behavioral-affective, and motor performance, including impaired verbal and nonverbal communication skills (e.g., language delays, reduced socially directed gaze/joint attention) and behavioral difficulties (e.g., poor emotion regulation and internalizing/externalizing problem behaviors).^1–4^ Moreover, they show motor incoordination, poor postural control, and poor socially embedded motor skills such as imitation and interpersonal synchrony.^5–9^

While contemporary interventions like Applied Behavior Analyses (ABA), Picture Exchange Communication System (PECS), and Teaching and Education of Autistic and Related Communication Handicapped Children (TEACCH) have been widely used to address ASD-related difficulties, they focus on sedentary activities devoid of whole-body movements.^10–12^ Creative movement (CM) interventions, on the other hand, emphasize whole-body movements and motor coordination, leading to positive effects on motor, social communication, and cognitive skills in children with ASD.^13–18^ During the COVID-19 pandemic, our research group delivered CM intervention face-to-face (F2F) or through telehealth (TH) to accommodate child and family preferences.^19–22^ In this article, we compare the fidelity, feasibility, acceptability, and efficacy of providing CM interventions for school-age children with ASD across F2F and TH delivery methods.

There is growing use of CM interventions (e.g., yoga, martial arts, dance, music) to address the multisystem challenges faced by children with ASD.^17^ These methods, which emphasize whole-body movements, are shown to have positive effects on motor, behavioral-affective, and social skills in individuals with ASD.^17^ A systematic review of 72 articles revealed medium-to-large-sized improvements in the motor and cognitive, as well as small-to-large-sized improvements in social communication skills in children with ASD following CM training.^17^ In addition, Arzoglou et al. reported improved balance and agility in children with ASD after 8 weeks of traditional Greek dance therapy.^23^

On the other hand, studies using musical/rhythm-based or yoga activities suggested improvements in gross motor coordination, imitation/praxis, and socially directed verbalization skills in children with ASD.^13–16,24,25^ These CM methods provide enjoyable and socially embedded contexts that challenge children's motor skills and encourage social information monitoring, verbal/nonverbal communication, and imitation/interpersonal synchrony.^2^ In this study, we developed a CM intervention combining music and yoga-based activities to target the multisystem impairments of children with ASD. In addition, we investigated how different delivery methods (F2F vs. TH) might impact children's outcomes within and outside the training context.

CM interventions have traditionally been delivered F2F; however, with advancements in communication technologies following the COVID-19 pandemic, remote delivery of therapies has gained popularity.^19,21,26^ In the past, TH has been a longstanding remote delivery method, providing access to services in the rural areas.^27,28^ While some survey studies suggest high parental satisfaction with receiving physical, occupational, and speech/language therapies when offered through TH, many families and health care providers still consider TH as a secondary option.^29,30^

Parents are concerned about playing an active role and supervising their child's TH sessions, while therapists are concerned about technological challenges and their ability to engage and manage children's behaviors through TH.^30^ In this study, we compared the feasibility, acceptability, and efficacy of providing CM interventions to children with ASD using F2F and TH methods. We hypothesized that children with ASD would demonstrate similar improvements in motor, behavioral-affective, and social communication skills following TH and F2F interventions, however, parents and trainers might report greater technological/communication challenges during TH compared to F2F CM interventions.

## Methods

### Participants

Fifteen children with ASD participated in the study (Age (average ± standard errors [SE]) = 9.4 ± 2.4; 12 males). Participants were recruited through local schools, ASD advocacy groups, and the Simons Powering Autism Research (SPARK) participant-research matching service (https://www.sfari.org/resource/spark/). We interviewed the parents to gather demographic information and confirm their eligibility for participation. Inclusion criteria consisted of an ASD diagnosis indicated by a school record confirmed from a school psychologist, and/or an Individualized Education Plan (IEP) for ASD-related services, and/or a medical/neuropsychological record from a psychiatrist or clinical psychologist using the Autism Diagnostic Observation Schedule (ADOS), and/or Autism Diagnostic Interview-Revised (ADI-R) measures. In addition, we used the Social Communication Questionnaire (SCQ) to screen for a social communication delay (Table 1).^31^

**Table 1.** Demographic Information and Data from Standardized Questionnaires Assessing Adaptive and Social Functioning in Face-to-Face and Telehealth Subgroups

Characteristics	*F2F group (*n* = 7), mean ± SE*	*TH group (*n* = 8), mean ± SE*	*Full group (*n* = 15), mean ± SE*
Age	9.7 ± 1.0	9.1 ± 0.8	9.5 ± 0.6
Sex	7M	5M, 3F	12M, 3F
Race	7C	4C, 3 AAC, 1A	10C, 1H, 3AAC, 1A
Ethnicity	7NH	1H, 7NH	1H, 14NH
SCQ	17.9 ± 2.2	16.0 ± 2.9	16.9 ± 1.8
VABS-II (SS)	71.4 ± 4.6	71.3 ± 4.0	71.4 ± 3.0
Communication (SS)	74.1 ± 4.6	75.5 ± 3.9	74.8 ± 3.5
Daily living (SS)	73.7 ± 5.3	73.0 ± 5.1	73.4 ± 3.5
Socialization (SS)	70.7 ± 5.4	69.7 ± 4.0	70.2 ± 3.3
SRS (T scores)	76.1 ± 4.5	79.7 ± 3.7	77.9 ± 2.9
SCI (T scores)	73.3 ± 3.7	78.0 ± 4.2	75.6 ± 2.8
RRB (T scores)	75.1 ± 4.1	83.7 ± 2.5	79.4 ± 2.6

Means and SE are provided. There was no significant difference between children in the F2F and TH subgroups on all variables (all *p* > 0.05).

RRB, Restricted Interests and Repetitive Behavior; SCI, Social Communication and Interaction; SCQ, Social Communication Questionnaire; SE, standard errors; SRS, Social Responsiveness Scale; SS, standard score; VABS-II, Vineland Adaptive Behavior Scale—2nd Edition; M, Male; F, Female; C, Caucasian; A, Asian; AA, African American; AAC, African American-Caucasian; H, Hispanic; NH, non-Hispanic.

The data reported in this article were part of a larger randomized controlled trial comparing the effects of creative and general movement interventions to a standard of care intervention. Due to COVID-19-related restrictions, we implemented a hybrid intervention model, offering the family the options of F2F or TH delivery method.^19–22^ Seven children received the CM intervention F2F, while the remaining eight children received through TH. No significant difference in age, sex, race, or ethnicity was observed between groups (*p* > 0.05, Table 1). Parents completed the Vineland Adaptive Behavioral Scales-Second Edition (VABS) and the Social Responsive Scale (SRS) questionnaire to assess their child's adaptive functioning and social communication skills (Table 1).^32,33^

Both children and parents provided written/verbal consent/assent for study participation and written permissions/consent to use their images for publication. This study was conducted as a collaborative effort between the University of Delaware and the University of Connecticut. The study procedures adhered to a single IRB protocol approved by the University of Delaware.

### Study procedures

We conducted pre-tests and post-tests before and after 8 weeks of CM intervention. During the testing sessions, we assessed the children's gross and fine motor performance. In addition, we analyzed videos from an early and a late training session to assess training-related changes in children's affective states, social verbalization, interpersonal synchrony, and motor coordination performance. Finally, we collected feedback from parents and trainers upon training completion.

### Training protocol for CM interventions

The CM intervention implemented musical/rhythm and yoga-based activities. We also incorporated treatment principles from conventional ASD approaches, including ABA, PECS, TEACCH, and motor learning theories, to provide structure/consistency and positive reinforcement, and allow children to engage in trial-and-error learning. We adapted our communication methods to each child's individual needs using picture schedules.

During training sessions, we divided the action sequences into parts, exaggerated key components, and offered clear visual and verbal instructions to facilitate learning. Positive reinforcement, such as high-fives and fist bumps, was utilized to motivate and acknowledge the child's performance. All children completed over 90% of the training sessions (number of completed training sessions [average ± SE]: F2F = 15.3 ± 0.2 and TH = 15.7 ± 0.2). There was no significant difference in training time between the F2F and TH subgroups (Training time in minutes [average ± SE]: F2F = 73.6 ± 5.6 and TH = 83.5 ± 5.1, *p* > 0.05).

**FIG. 1.**
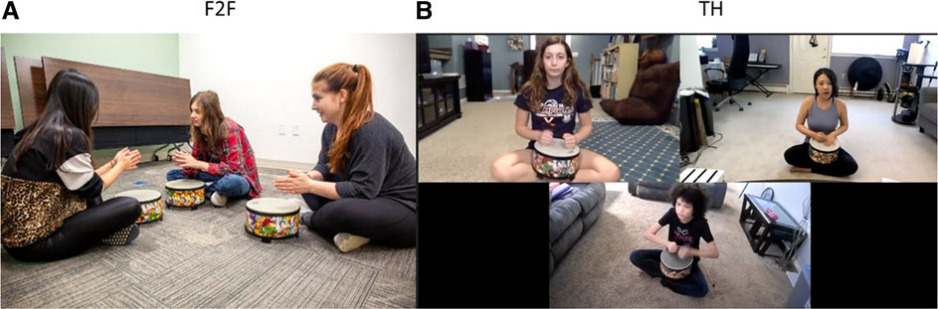
Picture examples for an F2F **(A)** and a TH **(B)** training session. F2F, face-to-face; TH, telehealth.

Each training session encompassed seven activities/conditions involving the child, trainer (trained graduate student/physical therapist), and a model (trained undergraduate student who acted as a buddy to the child), and/or parent. These conditions included *Hello Game, Action Game, Warm up, Music Game, Moving Game, Yoga & Breathing,* and *Farewell Song* (Fig. 1; Supplementary Table S1 and Supplementary Fig. S1). During an F2F session, the trainer and model stood/sat in a triadic arrangement with the child to demonstrate the activity, provide visual/verbal cues, and offer manual assistance (Fig. 1A). During a TH training session, the trainer and the model interacted with the child through a Zoom call (Fig. 1B). Parents were encouraged to prompt and move with the child to provide in-person demonstrations, cues, and assistance and to redirect the child's attention to the tasks.

### Testing or training-related measures

#### Standardized motor measures (pre-test and post-test)

We used the standardized Bruininks-Oseretsky Test of Motor Proficiency (BOT-2) and the Locomotor Subtest of the Test of Gross Motor Development-Second Edition (TGMD-2) to assess children's motor performance.^34,35^ BOT-2 is a valid and reliable assessment that includes four motor composites with eight subscales: (1) *Fine Manual Control* composite, consisting of the Fine Motor Precision and Fine Motor Integration subscales; (2) *Manual Coordination* composite, consisting of the Manual Dexterity and Upper-limb Coordination subscales; (3) *Body Coordination* composite, consisting of the Bilateral Coordination and Balance subscales; and (4) *Strength and Agility* composite, consisting of the Running Speed and Agility and Strength subscales.

On the other hand, the *locomotor subtest* of TGMD-2 assesses the quality of locomotor skill, including running, galloping, hopping, leaping, jumping, and sliding. The standard scores on all four composites of the BOT-2 and the locomotor subtest of the TGMD-2 were used to compare changes in motor performance from pre-test to post-test.

#### Behavioral coding during training sessions (early and late training)

Two trained student researchers used Datavyu to code children's affective states (percent duration in negative/noncompliant, neutral/off-task, interested/on-task, and positive/smiling affective states), socially directed verbalization (percent duration the child directed sounds or words toward a social partner), interpersonal synchrony (percent duration that child's actions were synchronized with the trainer/model), and motor coordination patterns (percent duration of single, dual, or multilimb actions).

For affective states, we combined the positive and interested affective states. *Social verbalization* was coded when the child used words or sounds to communicate with their social partners. *Interpersonal synchrony* was determined based on whether the child moved in a similar spatial manner and at the same time as the trainer/model (in-synchrony) or if they were unable to synchronize spatially and/or temporally (out of synchrony). Finally, *Motor coordination* was scored as “single,” “dual,” or “multilimb” depending on the number of limbs involved during each action sequence.

#### Stakeholder surveys

Before and after the intervention, we administered the Developmental Coordination Disorder Questionnaire (DCD-Q) to parents to understand their perceptions on their children's training-related motor improvements.^36^ In addition, we collected parent and trainer feedback on the feasibility, acceptability, and satisfaction with F2F versus TH delivery methods using online questionnaires. Parents provided feedback about the preparation time, efforts to engage their child, and their children's attention during the training sessions.

*Feasibility questions included the following*: (1) How demanding was the preparation time for the training sessions (travel, setup, etc.)? (2) How much effort did you put in to keep your child engaged during the training sessions? (3) How attentive was your child during the training sessions? *Acceptability questions included the following*: (1) How satisfied were you with the intervention delivery method (TH/F2F) your child received? (2) Was the intervention delivery method (TH/F2F) appropriate for your child? (3) Did your child benefit from the intervention delivery method (TH/F2F) he/she received? They were also asked about their overall satisfaction with the training sessions, and perceived benefits of the training. Finally, parents were asked to express their *preference for the method of training delivery* (F2F vs. TH) if the study were to be repeated in the future.

Trainer feedback was solicited regarding the feasibility of TH versus F2F delivery methods and any communication/technological issue they encountered during the sessions that impacted their interactions with the child/family. *Feasibility questions included*: (1) How easy was it for you to communicate with the child and parent during the training sessions? (2) What percentage of time was the parent involved in the training sessions? They were also asked to rate the challenges they encountered pertaining to session length and technological issues with F2F/TH methods. Parents and trainers rated each question using a five-point Likert score, which we later transferred into a three-point scale of feasible/satisfied, in between/neutral, and not feasible/not satisfied.

#### Training fidelity measure

We developed a training fidelity measure to examine the trainer's quality of providing instruction, prompt, and reinforcement during the training sessions. The fidelity coder selected one of the early and late training sessions and coded the trainer's behaviors toward the child (90 items, 141 points in total; Supplementary Table S2). The percent total, instructional, and prompt- and the reinforcement-related fidelity scores were calculated.

### Data analyses

For all standardized (i.e., BOT-2, TGMD-2, DCD-Q) and training-specific (i.e., positive/interested affect, social verbalization, interpersonal synchrony, and motor coordination patterns) measures, we present means, SE, and coefficients of variation (CV). Independent *t*-tests were used to compare between-group differences (F2F vs. TH) in training-related changes from pre-test/early to post-test/late sessions. For parent and trainer stakeholder surveys, we reported the percent respondents for each response category. Chi-square tests were used to compare the distribution of response categories between F2F and TH subgroups.

## Results

### Baseline comparisons

Upon comparing the baseline/pretest VABS or SRS scores between the F2F and TH subgroups, no significant between-group difference was seen (*p* > 0.05).

### Children's motor outcomes based on pre/post-testing

**FIG. 2.**
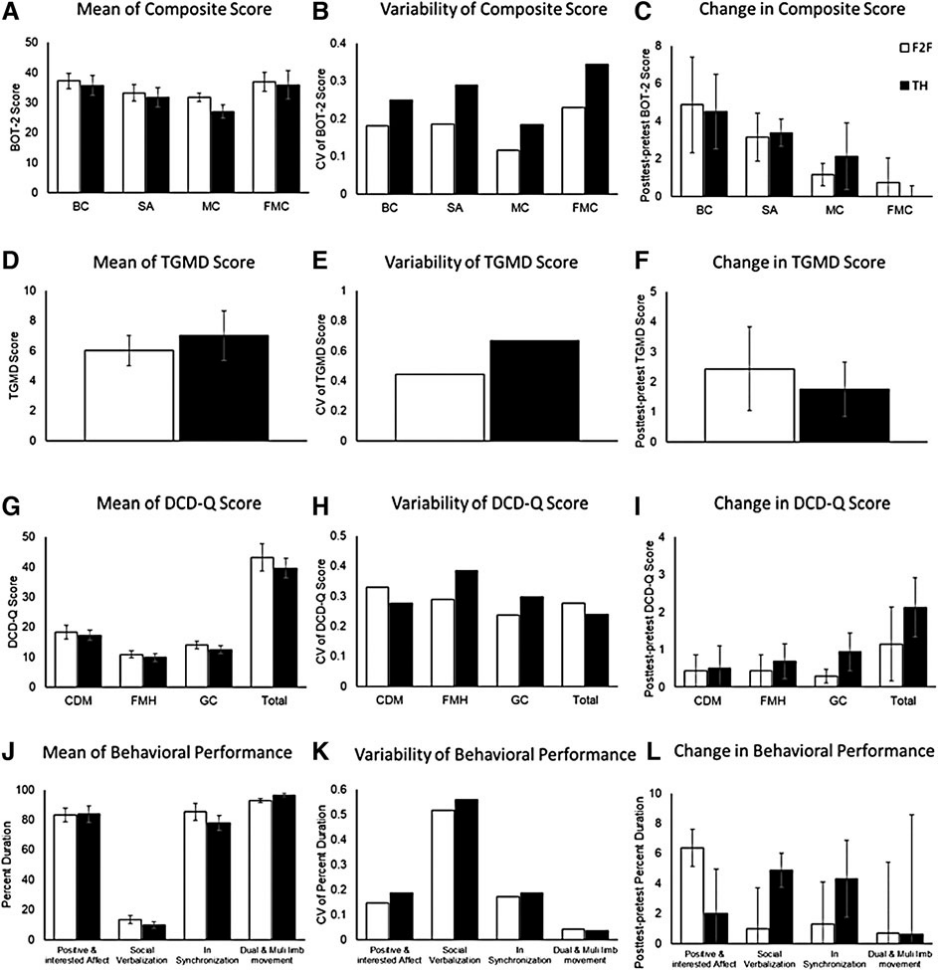
Mean, variability, and training-related changes in BOT-2 **(A–C)**, TGMD **(D–F)**, DCD-Q **(G–I)**, and behavioral performance **(J–L)**. BC, bilateral coordination; BOT-2, Bruininks Oseretsky Test of Motor Proficiency; CDM, Control During Movement; DCD-Q, Developmental Coordination Disorder Questionnaire; FMC, fine manual control; FMH, fine motor and handwriting; GC, general coordination; MC, Manual Coordination; SA, strength and agility; TGMD, Test of Gross Motor Development.

Children with ASD in both subgroups (F2F and TH) did not differ in mean and CV for BOT-2 and TGMD-2 scores at pre-test (*p* > 0.05; Fig. 2A, B, D, and E). Moreover, there was no difference in the magnitude of intervention-related improvements for all BOT-2 and the TGMD-2 measures between F2F and TH subgroups (*p* > 0.05; Fig. 2C, F). In terms of the DCD-Q questionnaire, parents in both subgroups reported similar mean and CV for DCD-Q scores at pre-test and similar magnitude of intervention-related improvements at post-test versus pre-test (*p* > 0.05; Fig. 2G–I; Supplementary Tables S3 and S4).

### Children's behavioral outcomes during training

The mean and CV of the duration of positive/interested affect, socially directed verbalization, interpersonal synchrony, and dual and multilimb motor coordination did not differ between the F2F and TH subgroups during the early training session (*p* > 0.05; Fig. 2J, K). Both subgroups did not differ in their training-related improvements in the percent duration of all behavioral measures (i.e., positive/interested affect, socially directed verbalization, interpersonal synchrony, and dual/multilimb motor coordination; *p* > 0.05; Fig. 2L; Supplementary Tables S3 and S4).

### Stakeholder feedback

#### Parent feedback

**FIG. 3.**
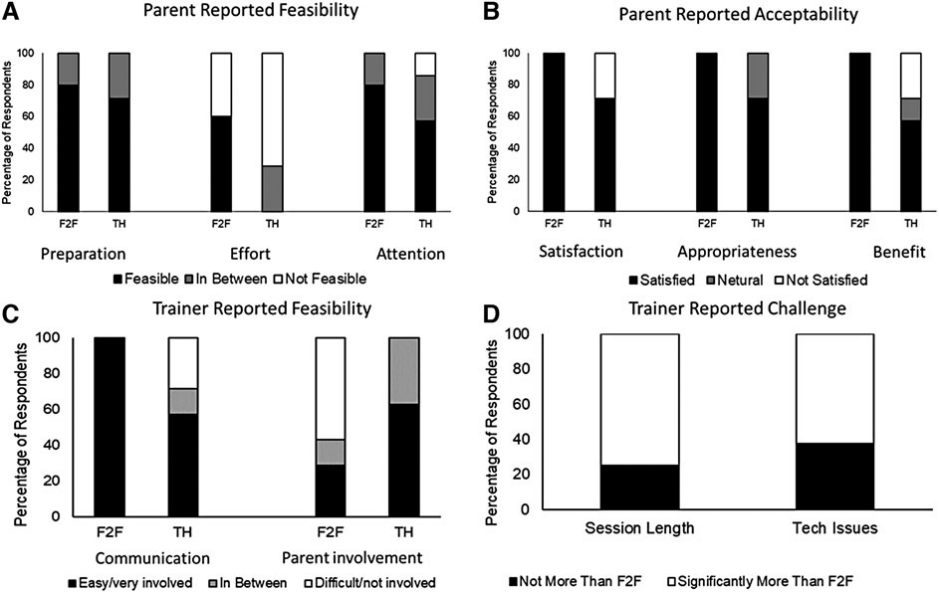
Parent- **(A, B)** and trainer- **(C, D)** reported feasibility and acceptability.

In terms of feasibility, perceived preparation time did not differ between subgroups [feasible preparation time (%): F2F: 80%; TH: 71%; *X*^2^ (2, *N* = 15) = 5.1, *p* > 0.05; Fig. 3A]. However, compared to the F2F subgroup, parents in the TH subgroup reported greater effort during the training sessions [feasible parental effort (%): F2F: 60%, TH: 0%, *X*^2^ (2, *N* = 15) = 25.1, *p* < 0.05; Fig. 3A].

In addition, compared to the F2F subgroup, parents in the TH subgroup reported lower attention during the training sessions [child's attending to tasks (%): F2F: 80%, TH: 57%; *X*^2^ (2, *N* = 15) = 71.4, *p* < 0.05; Fig. 3A]. In terms of acceptability, compared to the F2F subgroup, parents in the TH group found the sessions to be less satisfactory [satisfaction (%): F2F: 100%, TH: 71%; *X*^2^ (2, *N* = 15) = 37.9, *p* < 0.05; Fig. 3B], but appropriate [appropriate (%): F2F: 100%; TH: 71%; *X*^2^ (2, *N* = 15) = 37.9, *p* < 0.05; Fig. 3B] and beneficial [beneficial (%): F2F: 100%; TH: 57%; *X*^2^ (2, *N* = 15) = 70.5, *p* < 0.05; Fig. 3B] for their children.

#### Trainer feedback and fidelity

Compared to F2F, trainers reported greater communication challenges with TH delivery method [% trainers reporting feasible communication: F2F: 100%; TH: 57%; *X*^2^ (2, *N* = 15) = 70.5, *p* < 0.05; Fig. 3C]. However, trainers reported greater parental involvement with TH than F2F method [parental involvement (%): F2F: 28.6%; TH: 62.5%; *X*^2^ (2, *N* = 15) = 3167.9, *p* < 0.05; Fig. 3C].

In addition, trainers reported longer session times with TH (75% respondents) compared to F2F delivery (25% respondents; Fig. 3D); however, upon checking training fidelity, the actual session durations did not differ between F2F and TH subgroups. Only the reinforcement-related percent fidelity scores differed between the TH and F2F subgroups, with TH sessions involving slightly fewer reinforcements than the F2F sessions [Early: *t* (13) = 3.02, *p* < 0.05, Late: *t* (13) = 2.48, *p* < 0.05; Table 2]. Overall, in terms of fidelity, the session durations as well as the total session/prompt/instructional fidelity scores did not differ between the two subgroups (*p* > 0.05; Table 2).

**Table 2.** Training Session Length and the Percent Scores for Training Fidelity

	*F2F group (*n* = 7), mean (SE)*	*TH group (*n* = 8), mean (SE)*
Training session length (minute)	71.26 (5.18)	71.42 (4.84)
Fidelity total score (%)		
Early session	90.89 (1.17)	91.83 (1.65)
Late session	89.60 (2.14)	91.32 (2.77)
Prompts and instructions (%)		
Early session	90.04 (1.38)	91.80 (1.92)
Late session	88.82 (2.45)	92.27 (3.01)
Reinforcement (%)		
Early session	99.05 (0.95)^*^	88.83 (3.24)
Late session	95.92 (2.12)^*^	83.51 (4.59)

^*^ Significant differences between subgroups (*p* < 0.05).

## Discussion

Previous research has demonstrated the effectiveness of in-person CM interventions for individuals with ASD, with positive outcomes in social communication, behavioral-affective, and motor performance.^13–17^ However, due to the COVID-19 pandemic, many interventions have transitioned to TH delivery.^19–22,37^ In this study, we compared the acceptability, feasibility, and efficacy of F2F versus TH-based CM interventions using standardized motor assessments, training-specific measures, and stakeholder surveys completed by parents and trainers. Both F2F and TH subgroups showed similar baseline performance and training-related improvements in gross motor skills measured by standardized motor assessments (BOT-2 and TGMD) and a parent-report questionnaire (DCD-Q). Both subgroups showed comparable baseline performance and improvements in multiple behavioral variables such as positive/interested affect, socially directed verbalization, interpersonal synchrony, and motor coordination patterns.

Parents in the TH subgroup reported similar preparation time, but greater effort in supervising/redirecting their children's attention compared to parents in the F2F subgroup. Consequently, parents in the TH subgroup rated the training session less satisfying, but appropriate and beneficial for their children. Trainers also reported more communication/technological issues and longer session lengths with TH delivery, but acknowledged the positive aspect of greater parental involvement. In short, TH is an effective way for delivering CM intervention; however, clinicians should collaborate with families to address communication/technological challenges and alleviate parental burden when providing TH interventions.

### Comparable improvements in motor- and interpersonal synchrony-related outcomes in F2F and TH subgroups

Using standardized BOT-2 and TGMD-2 motor assessments and the DCD-Q parent questionnaire, we observed comparable training-related improvements in gross motor skills, including bilateral coordination, strength and agility, manual coordination, and locomotor skills among children receiving F2F and TH interventions. Our findings align with previous studies that reported gross motor improvements (i.e., increased BOT-2 gross motor scores and fewer imitation errors) following in-person music and yoga-based activities.^13,24,38^ Recent research also found that music-based interventions improve motor coordination and parent-reported DCD-Q scores in children with ASD.^38^ Improvements in motor performance may be attributed to the emphasis of whole-body coordination, strength and balance, and imitation/interpersonal synchrony.^2,13,17,24^ In this CM intervention, we incorporated these components into various conditions, including action songs, warm-up exercises, music making, moving games, and a yoga condition.

Such emphasis likely contributed to the observed enhancements in movement skills. Furthermore, we found it possible to engage children in music and yoga-based activities virtually. To promote engagement during TH interventions, we recommend using the gallery view to foster a sense of community, assigning the parent as the “therapist in the room” to provide manual support and move with the child, allowing children to observe the trainer/model closely before practicing the movements themselves, providing families with access to music/songs to play at their end, and using visual demonstrations to illustrate musical activities. Overall, with adequate preparation and support, our findings support the use of TH-based CM interventions to facilitate gross motor skills in children with ASD.

### Comparable levels of enjoyment and training-related improvements in socially directed verbalization between F2F and TH subgroups

We observed comparable improvements in behavioral-affective and social communication performance between the F2F and TH subgroups. Previous research also indicated positive effects of CM and physical activity interventions on the behavioral-affective performance of typically developing children and children with ASD.^17,39^ Physical activity has been proposed to release various neurochemicals, transmitters, and modulators (e.g., cortisol, dopamine, serotonin, and endogenous opioids) that contribute to better mood and behavioral regulation.^18,40^ The creativity and improvisation inherent to our activities may also have positive effects on affective/behavioral performance. For example, yoga and mindfulness interventions are known to regulate the sympathetic nervous system and hypothalamic-pituitary-adrenal system, leading to better emotional regulation.^18,41,42^

In this study, we used verbal and physical reinforcement (i.e., praises and physical/virtual high-five) to encourage/maintain children's engagement. Moreover, we tailored our training to the children's preferences by incorporating their favorite songs/characters/themes and encouraged them to create/choose their own moves, while retaining the key ingredients of the intervention. These efforts may contribute to the high engagement observed in both subgroups. Overall, both F2F and TH delivery methods for CM interventions are enjoyable and effective to promote engagement and behavioral regulation for children with ASD.

Similarly, we found improvements in socially directed verbalization in children from both subgroups. These findings align with previous studies that reported improved social communication skills in children with ASD following in-person music/rhythmic, and yoga-based interventions.^2,15,16,25,43^ A recent meta-analysis also suggested small-to-large-sized improvements in social communication skills following music and martial arts therapies.^17^ It is proposed that music and language share similar structure (both are hierarchically arranged) and engage overlapping neural substrates to process complex auditory information.^2,18,44^ The similarities between linguistic and musical experiences enable easy transfer between musical and communication skills.^2^ Moreover, music and movement activities naturally encourage spontaneous verbalization through singing, music making, and collective rhythmic activities with social partners.^2,15^

### Increased parental burden and reduced acceptability but greater parental involvement during TH versus F2F interventions

Parents in the TH subgroup reported similar preparation time, but greater effort in supervising and redirecting their child's attention compared to parents in the F2F subgroup. The substantial effort required on the part of TH parents may have contributed to their lower acceptability for virtual CM intervention delivery. Despite the lower acceptability rates, about 70% of TH parents (vs. 100% in the F2F subgroup) still found the intervention to be satisfactory, appropriate, and beneficial for their child, suggesting that TH remains an acceptable method for most families. Our findings align with a previous survey-based study that highlighted parental concerns about playing an active role during TH-based interventions.^30^ Compared to traditional seated play interventions like ABA, the CM intervention may pose greater challenges for parents in implementation, as it involved complex action sequences and physical movement to music.

We took steps to familiarize parents with their role and reviewed the training activities/movements beforehand. Nevertheless, unfamiliarity with the activities and reluctance to engage in whole-body movement may have made this intervention more challenging for the parent and the child. Although TH increases parental burden, it also facilitates parental involvement. Through active participation, parents gain awareness of the training activities and their child's difficulties.^20,21^ Previous studies on parent-mediated interventions suggested greater parental empowerment, reduced parental stress, and improved parental ability to support their child's learning after intervention completion.^45^ TH has the potential to positively influence child-caregiver interactions and foster interpersonal connections beyond the training context. Therefore, clinicians using CM approaches should strive to minimize parental burden/stress by choosing activities that families feel comfortable practicing, ensuring greater caregiver buy-in and participation.

### Greater communication/technological challenges expressed by clinicians during TH versus F2F interventions

Trainers faced more communication and technological issues with TH versus F2F interventions, leading to fewer reinforcements during TH sessions. Previous survey-based studies highlighted issues such as inadequate technologies/devices, limited user familiarity with videoconferencing, and poor internet connectivity during TH interventions.^45^ In our study, these communication issues and streaming delays were particularly crucial, given that music and interpersonal synchrony were key elements of the CM intervention. To overcome these issues, we provided families with step-by-step instructions for operating the technology and conducted pre-meetings with parents to set up videoconferencing software, optimized audio settings and camera angle, and identified a workspace with good internet connectivity and space for whole-body movements. In addition, we supplied parents with the music and training materials to reduce the audio lag as the child moved to music.

### Limitation and future directions

We acknowledge that our subgroup assignment to TH or F2F delivery method was not randomized. We also know that risk perception for COVID-19 infection differed between the TH and F2F families as they self-selected those delivery formats. Therefore, future studies, including large samples that employ randomized allocation to delivery methods, are needed. While the trainers in the study were equally assigned to the TH and F2F subgroups, models/supporting adults (parent or trained undergraduate students) varied across participants and may have contributed to data variability. Note that this study aimed to compare outcomes, fidelity, and feasibility of CM intervention delivered in F2F versus TH format, whereas a future publication will report the training effects of CM intervention in comparison to a sedentary play, control intervention.

## Conclusions

Both TH and F2F subgroups demonstrated comparable improvements in gross motor skills, interpersonal synchrony, behavioral-affective performance, and socially directed verbalizations following CM interventions. Despite the greater burden reported by parents and communication/technological issues reported by trainers in the TH subgroup, this method yielded positive effects in motor, affective, and social communication domains in children with ASD. In addition, TH facilitates active parental involvement during the intervention. Clinicians delivering TH intervention should be aware of the associated communication and technological challenges, receive training to address them, and should provide support to families in overcoming such issues before and during the training sessions.

## Supplementary Material

Supplemental data

Supplemental data

Supplemental data

Supplemental data

Supplemental data

## Abbreviations Used

ABA: Applied Behavior Analyses
ASD: autism spectrum disorder
BOT-2: Bruininks Oseretsky Test of Motor Proficiency-Second Edition
CM: creative movement
CV: coefficients of variation
DCD-Q: Developmental Coordination Disorder Questionnaire
F2F: face-to-face
PECS: Picture Exchange Communication System
SE: standard errors
SPARK: Simons Powering Autism Research
SRS: Social Responsive Scale
TEACCH: Teaching and Education of Autistic and Related Communication Handicapped Children
TGMD-2: Test of Gross Motor Development-Second Edition
TH: telehealth
VABS: Vineland Adaptive Behavioral Scales-Second edition
