# Supplementary material for: A Pilot Study Comparing the Efficacy, Fidelity, Acceptability, and Feasibility of Telehealth and Face-to-Face Creative Movement Interventions in Children with Autism Spectrum Disorder
Source: Telemed Rep. 2024 Mar 21;5(1):67–77. doi: 10.1089/tmr.2023.0061 (PMC10979681; doi:10.1089/tmr.2023.0061)
Supplement: Supplemental data [file Suppl_TableS1.docx]

**Supplementary Table S1. Conditions, examples, and targeted skills during CM intervention**

| Condition | ExamplAR activities | Targeted Motor skills | Other skills |
| --- | --- | --- | --- |
| 1. Hello Game | Hot potato: Pass the bean bag around when the music is on, when the music stops, the person who has the bean bag answers a question asked by another. | Bimanual coordination, sensorimotor integration | Social communication, executive functioning |
| 1. Action Game | Peter Pan: Sing and play hand games/action sings with partners | Bimanual coordination, sensorimotor integration, imitation, IPS | Social communication |
| 1. Warm up | Birthday party: Stretch and use different props (i.e., pool noodles, scarves) to provide sensory inputs on different body parts, e.g., tapping, brushing, etc. | Multi-limb coordination, flexibility, movement improvisation | Sensory processing |
| 1. Music Game | Stop and Go: Sing and perform whole-body movements with musical instruments (i.e., Maracas, cymbals).  Drum circle: Drum synchronously and/or play call and response games (requires turn taking) with the trainer or model | Whole body and bimanual coordination, imitation, IPS | Executive functioning, social communication |
| 1. Moving Game | Move and Freeze: When the music starts, move around using one of the locomotor skills. When the music stops, freeze on a yoga spot and maintain balancing poses. | Locomotor skills (i.e., running, galloping, hopping, leaping, jumping, sliding, skipping), balance, strength and endurance, imitation, IPS | Executive functioning, social communication |
| 1. Yoga & Breathing | Yoga story: Do yoga poses according to a storyline (e.g., Luke’s beach day). Bird breath: Breathing in and out while moving arms up and down. | Balance, strength and endurance, flexibility, imitation, IPS | Mindfulness, reading, social communication |
| 1. Farewell | So long, farewell: Sing, reflect, and say goodbye | Movement improvisation | Social communication |
