# Supplementary material for: A Pilot Study Comparing the Efficacy, Fidelity, Acceptability, and Feasibility of Telehealth and Face-to-Face Creative Movement Interventions in Children with Autism Spectrum Disorder
Source: Telemed Rep. 2024 Mar 21;5(1):67–77. doi: 10.1089/tmr.2023.0061 (PMC10979681; doi:10.1089/tmr.2023.0061)
Supplement: Supplemental data [file Suppl_FigureS1.docx]

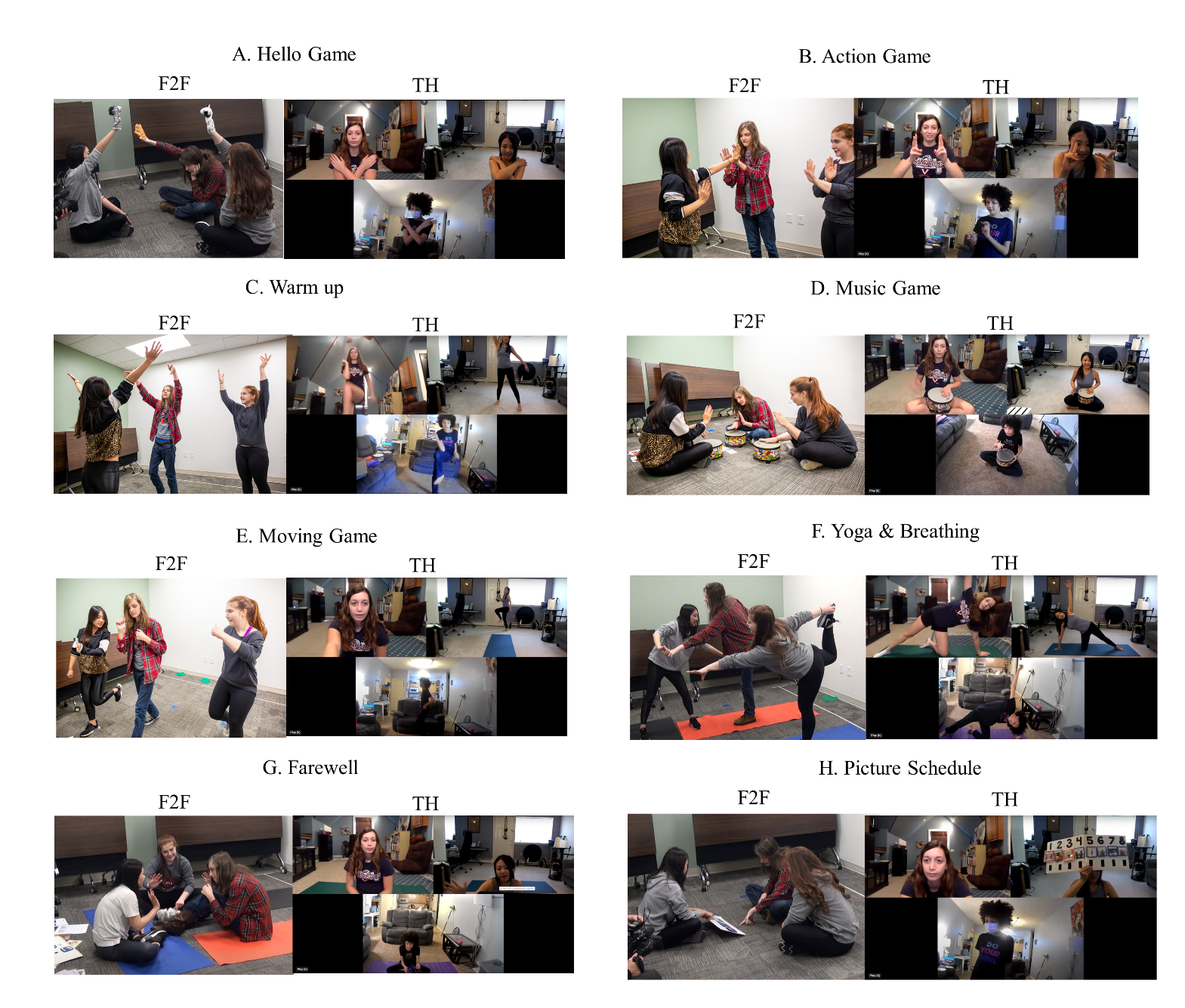
**Supplementary Figure S1.** Pictures of F2F and TH training activities, including the Hello Game (A), Action Game (B), Warm up (C), Music Game (D), Moving Game (E), Yoga & Breathing (F), and Farewell (G). The Picture schedule is also presented (H). Written permission for publication of participant pictures has been taken.
