# Supplementary material for: A Pilot Study Comparing the Efficacy, Fidelity, Acceptability, and Feasibility of Telehealth and Face-to-Face Creative Movement Interventions in Children with Autism Spectrum Disorder
Source: Telemed Rep. 2024 Mar 21;5(1):67–77. doi: 10.1089/tmr.2023.0061 (PMC10979681; doi:10.1089/tmr.2023.0061)
Supplement: Supplemental data [file Suppl_TableS2.docx]

**Supplementary Table S2.** Fidelity checklist for the Creative Movement group

| **Training Components** | **Explanation** | **Maximum points (173)** | **Score** | **Comments** |  |
| --- | --- | --- | --- | --- | --- |
| **Hello Game** | | | | |  |
| Show PECS to the child. | Trainer shows the PECS and introduces all the activities for the day. | 1 point |  |  |  |
| Introduce condition using PECS | Trainer asks the child, “So what are we going to do first? Can you point to it/tell me?” | 1 point |  |  |  |
| Ready position & Ready response | Trainer ensures the child is on his/her spot, receives a ready response, and asks "Are you ready to say hello to everyone?" | 1 point |  |  |  |
| Hello Game Instructions | Trainer says, “Today we are going to sing the XYZ song/ play the XYZ game”. | 1 point |  |  |  |
| Request Help with Getting Props (Only if needed) | Asks child to help get props for activity | 1 point |  |  |  |
|  |  |  |  |  |  |
|  |  |  |  |  |  |
|  |  |  |  |  |  |
| Hello Game Trials | **When singing hello songs:** - 1st trial – Trainer sings the song and says hi to the child. - 2nd trial – Trainer waits for the child to fill in words during the song and asks the child to say hi to the model.  - 3rd trial – Trainer waits for the child to fill in words during the song and asks the child to say hi to the trainer. **When Playing Hello Games:** - 1st trial – Play the game together. Model introduces themselves and asks the child a question (Social bid). - 2nd trial – Play the game together. Child introduces themselves and asks a question to the model/trainer. - 3rd trial – Play the game together. Trainer introduces themselves and asks the child a question. | 3 points (1 point per trial) |  |  |  |
| Social Praise 1-1 | **Gestural:** Model provides hi-fives, low-fives, fist bumps, and waits for a response (at least 1 bid). **Verbal:** Trainer provides verbal reinforcement (at least 1 bid). | 2 points (1 point for trainer bid and 1 point for model bid) |  |  |  |
| Request Help for returning Props (Only if needed) | Trainer asks the child to help put props away at the end of the activity. | 1 point |  |  |  |
|  |  |  |  |  |  |
|  |  |  |  |  |  |
|  |  |  |  |  |  |
| **Finger Game/Action Song** | | | | |  |
| Hand Game Instructions | Trainer says, “Today we are going to do the XYZ hand game. | 1 point |  |  |  |
| Request Help with Getting Props (Only if needed) | Trainer asks the child to help get props for activity. | 1 point |  |  |  |
|  |  |  |  |  |  |
|  |  |  |  |  |  |
|  |  |  |  |  |  |
| Hand Gesture Trials | - 1^st^ trial – Trainer sings and gestures to the song and encourages the child to follow. | 2 points (1 point per trial) |  |  |  |
|  | - 2^nd^ trial – Trainer waits for the child to make the gestures/moves while singing the song. |  |  |  |  |
| Social Praise 1-2 | **Gestural:** Model provides hi-fives, low-fives, fist bumps, and waits for a response (at least 1 bid). **Verbal:** Trainer provides verbal reinforcement (at least 1 bid). | 2 points (1 point for trainer bid and 1 point for model bid) |  |  |  |
| Social bid 1 | Model makes the social bid, waits for the child to answer, and then makes an appropriate follow-up comment. | 1 point |  |  |  |
| Request for help to clean up | Trainer asks the child to help put props away at the end of the activity. | 1 point |  |  |  |
| Quality of Prompts - Appropriateness | Trainer/model provides appropriate visual, verbal, gestural, and manual prompts. | 4 points (1 for visual, verbal, gestural, and manual) |  |  |  |
| Transition with PECS | Trainer prompts the child to move the picture down on the board following the completion of the condition. | 1 point |  |  |  |
| **Warm up** | | | | |  |
| Introduce condition using PECS | Trainer asks the child, “So what are we going to do next? Can you point to it/tell me?”. | 1 point |  |  |  |
| Ready position & Ready response | Trainer ensures the child is on his/her spot, receives a ready response, and asks "Are you ready to play the warm up game?". | 1 point |  |  |  |
| Condition Instructions | Trainer says, “Today we are going to play the XYZ game. | 1 point |  |  |  |
| Request Help with Getting Props (Only if needed) | Trainer asks the child to help get props for activity. | 1 point |  |  |  |
|  |  |  |  |  |  |
|  |  |  |  |  |  |
| Trials (1trial only, if child does not do anything in trial 1, then repeat for trial 2) | - 1^st^ trial – Trainer leads the game and motivates the child to participate in the game. | 2 points (0- follow<25%; 1- 25~50%; 2:>50%) |  |  |  |
|  | - Repeat the trial only if the child doesn’t follow. |  |  |  |  |
| Social praise 2-1– Gestural & Verbal | **Gestural:** Model provides hi-fives, low-fives, or fist bumps and waits for a response (at least 1 bid). **Verbal:** Trainer provides verbal reinforcement (at least 1 bid). | 2 points (1 point for trainer bid and 1 point for model bid) |  |  |  |
| Social bid 2 | Model makes the social bid, waits for the child to answer, and then makes an appropriate follow-up comment. | 1 point |  |  |  |
| Request for help to clean up | Trainer asks the child to help put props away at the end of the activity. | 1 point |  |  |  |
| Quality of Rhythmic Synchrony/Speed | Trainer and Model move at an appropriate pace that the child can follow and wait for the child to join the group for each new move. | 1 point |  |  |  |
| Quality of Prompts - Appropriateness | Trainer/model provides appropriate visual, verbal, gestural, and manual prompts. | 4 points (1 for visual, verbal, gestural, and manual) |  |  |  |
| Quality of Skills Training | Trainer shows the activity, provides an accurate visual for the child to follow, and gives opportunities for the child to practice on his/her own. | 2 points, 0 = not at all, 1 = part of condition 2 = throughout entire condition |  |  |  |
| Transition with PECS | Trainer prompts the child to move the picture down on the board following the completion of the condition. | 1 point |  |  |  |
| **Music Time** | | | | |  |
| Introduce condition using PECS | Trainer asks the child, “So what are we going to do next? Can you point to it/tell me?” | 1 point |  |  |  |
| Ready position & Ready response | Trainer ensures the child is on his/her spot, receives ready response, and asks "Are you ready for the music time?". | 1 point |  |  |  |
| Condition Instructions | Trainer says, “It’s music time. Are you ready to play with XYZ instrument?” | 1 point |  |  |  |
| Request Help with Getting Props | Trainer asks the child to help get props for activity. | 1 point |  |  |  |
|  |  |  |  |  |  |
|  |  |  |  |  |  |
| Trials | Prompted trial 1 – Trainer provides verbal prompts or hand on hand assistance if the child does not follow the pattern. Score full points if the child can perform the pattern without assistance.  Unprompted trial 2 – Trainer does not provide any kind of prompts for this trial. Allows free movement. | 2 points (1 point per trial) |  |  |  |
| Instructions for mirroring | Trainer encourages the child to mirror his/her actions by saying “Make sure to copy me, If I use, right hand, which hand will you use?” | 1 point |  |  |  |
| Social praise 3-1– Gestural & Verbal | **Gestural**: Model provides hi-fives, low-fives, fist bumps, and waits for a response (at least 1 bid).  **Verbal:** Trainer provides verbal reinforcement (at least 1 bid). | 2 points (1 point for trainer bid and 1 point for model bid) |  |  |  |
| Request for help to clean up | Trainer asks the child to help put props away at the end of the activity. | 1 point |  |  |  |
| Drum Condition Instructions | Trainer says, “It’s drumming time. Are you ready to drum with us?” | 1 point |  |  |  |
| Request Help with Getting Props | Trainers asks the child to help get props for activity and asks the child to put props away at the end of the activity. | 2 points (1 for getting props, 1 for returning props) |  |  |  |
| Drum Trial | Rumbles – freely drums and encourages everyone to do so. | 1 point |  |  |  |
| Drum Trial | Sings and drums to the rhythm of the theme song for the day. | 1 point |  |  |  |
| Drum Trial | Choose instruction cards. | 1 point |  |  |  |
| Drum Trial | Model arranges the cards and ask the child to play the beats together. | 1 point |  |  |  |
| Drum Trial | Trainer asks the child to arrange cards and play the syllables/beats with the group. | 1 point |  |  |  |
| Drum Trial | Turn taking: Trainer asks the question, Model chooses the answer and plays the syllables/beats with the child. | 1 point |  |  |  |
| Drum Trial | Turn taking: Trainer asks the question; child chooses the answer and plays the beats with the Model. | 1 point |  |  |  |
| Social praise 3-2 – Gestural & Verbal | **Gestural:** Model provides hi-fives, low-fives, fist bumps, and waits for response (at least 1 bid). **Verbal:** Trainer provides verbal reinforcement (at least 1 bid). | 2 points (1 for trainer bid and 1 for model bid) |  |  |  |
| Request for help to clean up | Trainer asks the child to help put props away at the end of the activity. | 1 point |  |  |  |
| Quality of Rhythmic Synchrony/Speed | Trainer and Model move at an appropriate pace that the child can follow and waits for child to join the group for each new move. | 1 point |  |  |  |
| Quality of Prompts – Appropriateness | Trainer/model provides appropriate visual, verbal, gestural, and manual prompts. | 4 points (1 for visual, verbal, gestural, and manual) |  |  |  |
| Quality of Skills Training | Trainer shows the activity, provides an accurate visual for the child to follow, gives opportunities for the child to practice on his/her own. | 2 points, 0 = not at all, 1 = part of condition 2 = throughout entire condition |  |  |  |
| Social bid 3 | Model makes the social bid, waits for the child to answer, and then makes an appropriate follow-up comment. | 1 point |  |  |  |
| Transition with PECS | Trainer prompts the child to move the picture down on the board following the completion of the condition. | 1 point |  |  |  |
| **Moving game** | | | | |  |
| Introduce condition using PECS | Trainer asks the child, “So what are we going to do next? Can you point to it/tell me?” | 1 point |  |  |  |
| Ready position & Ready response | Trainer ensures the child is on his/her spot, receives ready response, and asks “Are you ready for the moving game?”. | 1 point |  |  |  |
| Condition Instructions | Trainer says, “Today we are going to play the XYZ game.” | 1 point |  |  |  |
| Request Help with Getting Props (Only if needed) | Trainer asks the child to help get the props for the activity. | 1 point |  |  |  |
| Movement Instruction | Trainer tells the child how the activity will be done and reviews any movements that the child may need to practice before performing them, Emphasizing form of the movements using keywords/demonstrations (e.g., step-hop for skipping or drag feet for sliding, etc.). | 1 point |  |  |  |
| Social praise 4-1 – Gestural & Verbal | **Gestural:** Model provides hi-fives, low-fives, fist bumps, and waits for a response (at least 1 bid). **Verbal:** Trainer provides verbal reinforcement (at least 1 bid). | 2 points (1 for trainer bid and 1 for model bid) |  |  |  |
| Trials | The child completes 2 trials of the activity. | 2 points (1 per trial) |  |  |  |
| Social praise 4-2– Gestural & Verbal | **Gestural:** Model provides hi-fives, low-fives, fist bumps, and waits for a response (at least 1 bid). **Verbal:** Trainer provides verbal reinforcement (at least 1 bid). | 2 points (1 for trainer bid and 1 for model bid) |  |  |  |
| Request for help to clean up | Trainer asks the child to help put the props away at the end of the activity. | 1 point |  |  |  |
| Quality of Rhythmic Synchrony/Speed | Trainer/Model moves at an appropriate pace that the child can follow and waits for child to join the group for each new move. | 1 point |  |  |  |
| Quality of Prompts – Appropriateness | Trainer/model provides appropriate visual, verbal, gestural, and manual prompts. | 4 points (1 for visual, verbal, gestural, and manual) |  |  |  |
| Quality of Skills Training | The trainer shows the activity, provides an accurate visual for the child to follow, and gives opportunities for the child to practice on his/her own. | 2 points, 0 = not at all, 1 = part of condition 2 = throughout entire condition |  |  |  |
| Transition with PECS | Trainer prompts the child to move the picture down on the board following the completion of the condition. | 1 point |  |  |  |
| **Yoga** | | | | |  |
| Introduce condition using PECS | Trainer asks the child, “So what are we going to do next? Can you point to it/tell me?” | 1 point |  |  |  |
| Ready position & Ready response | Trainer ensures the child is on his/her spot, receives a ready response, and asks “Are you ready to do yoga with us?”. | 1 point |  |  |  |
| Request Help with Getting Props | Trainer asks the child to help get props for the yoga activity. | 1 point |  |  |  |
| Condition Instructions | Trainer says, “Today we are going to practice poses on story/song/game. Copy me and do as I do”. | 1 point |  |  |  |
| Practice the poses | Trainer says, “Let’s practice the poses first.” The child completes 4 poses, holds 10 seconds for each pose. | 4 points (1 point per pose) |  |  |  |
| Social praise 5-1– Gestural & Verbal | **Gestural:** Model provides hi-fives, low-fives, fist bumps, and waits for a response (at least 1 bid). **Verbal:** Trainer provides verbal reinforcement (at least 1 bid). | 2 points (1 point for trainer bid and 1 point for model bid) |  |  |  |
| Trials - music/story | Trainer asks the child to dance/do appropriate gestures while singing the yoga song/telling the yoga story. | 2 points (0-Never follow; 1-follow 25-50%; 2: follow>50%) |  |  |  |
| Trials - poses | The child completes 4 poses: Hold 10 seconds for each pose. Change side when applicable. | 4 points (1 point per pose) |  |  |  |
| Social praise 5-2– Gestural & Verbal | **Gestural:** Model provides hi-fives, low-fives, fist bumps, and waits for a response (at least 1 bid). **Verbal:** Trainer provides verbal reinforcement (at least 1 bid). | 2 points (1 point for trainer bid and 1 point for model bid) |  |  |  |
| Quality of Prompts – Appropriateness | Trainer/model provides appropriate visual, verbal, gestural, and manual prompts. | 4 points (1 for visual, verbal, gestural, and manual) |  |  |  |
| Quality of Skills Training | Trainer shows the activity, provides an accurate visual for the child to follow, and gives opportunities for the child to practice on his/her own. | 2 points, 0 = not at all, 1 = part of condition 2 = throughout entire condition |  |  |  |
| Transition with PECS | Trainer prompts the child to move the picture for the condition down on the board following the completion of the condition. | 1 point |  |  |  |
| **Breathing** | | | | |  |
| Introduce condition using PECS | Trainer asks the child, “So what are we going to do next? Can you point to it/tell me?” | 1 point |  |  |  |
| Ready position & Ready response | Trainer ensures the child is on his/her spot, receives a ready response, and asks “Are you ready to take some deep breaths and relax?”. | 1 point |  |  |  |
| Request Help with Getting Props (if props used) | The trainer asks the child to help get props for the breathing activity. | 1 point |  |  |  |
| Demonstrate breathing | Trainer says, “today we’re going to practice XYZ breath.” “Copy me and do as I do.” | 1 point |  |  |  |
| Breathing trial | All participants repeat the breathing activity 3-5 times. | 3 points (1point per trial) |  |  |  |
| Social praise – Gestural & Verbal | **Gestural:** Model provides hi-fives, low-fives, fist bumps, and waits for a response (at least 1 bid). **Verbal:** Trainer provides verbal reinforcement (at least 1 bid). | 2 points (1 point for trainer bid and 1 point for model bid) |  |  |  |
| Request for help to clean up | Trainer asks the child to help put the props away at the end of the activity. | 1 point |  |  |  |
| Quality of Prompts - Appropriateness | Trainer/model provides appropriate visual, verbal, gestural, and manual prompts | 4 points (1 for visual, verbal, gestural, and manual) |  |  |  |
| Quality of Skills Training | Trainer shows the activity, provides an accurate visual for the child to follow, and gives opportunities for the child to practice on his/her own. | 2 points, 0 = not at all, 1 = part of condition 2 = throughout entire condition |  |  |  |
| Transition with PECS | Trainer prompts the child to move the picture for the condition down on the board following the completion of the condition. | 1 point |  |  |  |
| **Farewell** | | | | |  |
| Reflexional social bid | Model askes: “What was your favorite activity” “What’s was your favorite move?” “What’s your favorite pose?” | 3 points (1 point per question) |  |  |  |
| Introduce condition using PECS | Trainer asks the child, “So what are we going to do last? Can you point to it/tell me?” | 1 point |  |  |  |
| Ready position & Ready response | Trainer ensures the child is on his/her spot, receives a ready response, and asks "Are you ready to sing the goodbye song?". Group bids goodbye to each other while singing the song. | 1 point |  |  |  |
