# Supplementary material for: A Pilot Study Comparing the Efficacy, Fidelity, Acceptability, and Feasibility of Telehealth and Face-to-Face Creative Movement Interventions in Children with Autism Spectrum Disorder
Source: Telemed Rep. 2024 Mar 21;5(1):67–77. doi: 10.1089/tmr.2023.0061 (PMC10979681; doi:10.1089/tmr.2023.0061)
Supplement: Supplemental data [file Suppl_TableS3.docx]

**Supplementary Table S3.** Mean, SE, and between-group t-statistics for motor and behavioral performance during pretest/ early training session.

|  | **F2F subgroup**  **(n = 7)** | | **TH subgroup**  **(n = 8)** | | **Between-group t-statistics** | | |
| --- | --- | --- | --- | --- | --- | --- | --- |
|  | **Mean** | **SE** | **Mean** | **SE** | **t** | **df** | **p-value** |
| **BOT-2, BC (SS)** | 37.14 | 2.55 | 35.71 | 3.36 | 0.34 | 12 | 0.74 |
| **BOT-2, SA (SS)** | 33.20 | 2.75 | 31.75 | 3.23 | 0.31 | 11 | 0.76 |
| **BOT-2, MC (SS)** | 31.71 | 1.39 | 27.00 | 2.21 | 1.90 | 10 | 0.09 |
| **BOT-2, FMC (SS)** | 36.86 | 3.19 | 35.86 | 4.67 | 0.18 | 12 | 0.86 |
| **TGMD (SS)** | 6.00 | 1.00 | 7.00 | 1.65 | -0.50 | 13 | 0.63 |
| **DCD-Q, CDM** | 18.29 | 2.28 | 17.25 | 1.69 | 0.37 | 13 | 0.72 |
| **DCD-Q, FMH** | 10.86 | 1.18 | 9.88 | 1.34 | 0.54 | 13 | 0.60 |
| **DCD-Q, GC** | 14.00 | 1.25 | 12.50 | 1.31 | 0.82 | 13 | 0.43 |
| **DCD-Q, Total** | 43.14 | 4.50 | 39.63 | 3.34 | 0.64 | 13 | 0.53 |
| **Positive & Interested Affect (%)** | 83.28 | 4.61 | 83.83 | 5.53 | -0.76 | 13 | 0.94 |
| **Social verbalization (%)** | 13.40 | 2.61 | 9.80 | 2.07 | 1.08 | 12 | 0.30 |
| **In synchrony (%)** | 85.29 | 5.55 | 77.84 | 5.13 | 0.99 | 13 | 0.34 |
| **Dual & Multi limb movements (%)** | 92.80 | 1.46 | 96.41 | 1.20 | -1.92 | 13 | 0.77 |

BOT-2 = Bruininks-Oseretsky Test of Motor Proficiency, second edition; TGMD-2 = Test of Gross Motor Development, second edition; DCD-Q = Developmental Coordination Disorder Questionnaire; BC = Body Coordination; SA = Strength and Agility; MC = Manual Coordination; FMC = Fine Manual Control; CDM = Control during movement; FMH = Fine motor and handwriting; GC = general coordination; SS = Standard Score; F2F = Face to Face; TH = Telehealth.
