# Supplementary material for: A Pilot Study Comparing the Efficacy, Fidelity, Acceptability, and Feasibility of Telehealth and Face-to-Face Creative Movement Interventions in Children with Autism Spectrum Disorder
Source: Telemed Rep. 2024 Mar 21;5(1):67–77. doi: 10.1089/tmr.2023.0061 (PMC10979681; doi:10.1089/tmr.2023.0061)
Supplement: Supplemental data [file Suppl_TableS4.docx]

**Supplementary Table S4.** The effect sizes (Hedge’s g) and the between-group t-statistics of the training-related improvements.

|  | **F2F subgroup**  **(n = 7)** | | **TH subgroup**  **(n = 8)** | | **Between-group t-statistics** | | |
| --- | --- | --- | --- | --- | --- | --- | --- |
|  | **Hedge’s g** | **95%CI** | **Hedge’s g** | **95%CI** | **t** | **df** | **p-value** |
| **BOT-2, BC (SS)** | 0.63 | -0.42~1.67 | 0.48 | -0.41~1.37 | 0.00 | 6 | 1.00 |
| **BOT-2, SA (SS)** | 0.53 | -0.48~1.53 | 0.32 | -0.52~1.18 | -0.51 | 6 | 0.63 |
| **BOT-2, MC (SS)** | 0.27 | -0.66~1.21 | 0.48 | -0.41~1.37 | -0.81 | 6 | 0.45 |
| **BOT-2, FMC (SS)** | 0.07 | -0.84~0.98 | -0.01 | -0.83~0.81 | 0.51 | 6 | 0.63 |
| **TGMD (SS)** | 0.80 | -0.32~1.92 | 0.33 | -0.52~1.19 | 0.12 | 6 | 0.91 |
| **DCD-Q, CDM** | 0.06 | -0.85~0.97 | 0.09 | -0.73~0.92 | -0.19 | 6 | 0.86 |
| **DCD-Q, FMH** | 0.12 | -0.79~1.03 | 0.16 | -0.67~0.99 | -0.47 | 6 | 0.66 |
| **DCD-Q, GC** | 0.08 | -0.93~0.99 | 0.23 | -0.61~1.06 | -1.36 | 6 | 0.22 |
| **DCD-Q, Total** | 0.08 | -0.83~0.99 | 0.20 | -0.63~1.03 | -1.08 | 6 | 0.32 |
| **Positive & Interested Affect (%)** | 0.45 | -0.53~1.44 | 0.11 | -0.71~0.94 | 1.02 | 6 | 0.35 |
| **Social verbalization (%)** | 0.12 | -0.79~1.04 | 0.84 | -0.18~1.86 | -1.56 | 6 | 0.17 |
| **In synchrony (%)** | 0.08 | -0.83~0.99 | 0.26 | -0.58~1.10 | -0.54 | 6 | 0.61 |
| **Dual & Multi limb movements (%)** | 0.16 | -0.76~1.08 | 0.16 | -0.66~0.99 | -0.39 | 6 | 0.71 |

BOT-2 = Bruininks-Oseretsky Test of Motor Proficiency, second edition; TGMD-2 = Test of Gross Motor Development, second edition; DCD-Q = Developmental Coordination Disorder Questionnaire; BC = Body Coordination; SA = Strength and Agility; MC = Manual Coordination; FMC = Fine Manual Control; CDM = Control during movement; FMH = Fine motor and handwriting; GC = general coordination; SS = Standard Score; F2F = Face to Face; TH = Telehealth.
